# Behavioral and electrophysiological evidence for a neuroprotective role of aquaporin-4 in the 5xFAD transgenic mice model

**DOI:** 10.1186/s40478-020-00936-3

**Published:** 2020-05-12

**Authors:** Yoichiro Abe, Natsumi Ikegawa, Keitaro Yoshida, Kyosuke Muramatsu, Satoko Hattori, Kenji Kawai, Minetaka Murakami, Takumi Tanaka, Wakami Goda, Motohito Goto, Taichi Yamamoto, Tadafumi Hashimoto, Kaoru Yamada, Terumasa Shibata, Hidemi Misawa, Masaru Mimura, Kenji F. Tanaka, Tsuyoshi Miyakawa, Takeshi Iwatsubo, Jun-ichi Hata, Takako Niikura, Masato Yasui

**Affiliations:** 1 Department of Pharmacology, Keio University School of Medicine, 35 Shinanomachi, Shinjuku-ku, Tokyo, 160-8582 Japan; 2 Center for Water Biology & Medicine, Keio University Global Research Institute, Mita, Tokyo, 108-8345 Japan; 3 Department of Information and Communication Sciences, Faculty of Science and Technology, Sophia University, 7-1 Kioi-cho, Chiyoda-ku, Tokyo, 102-8554 Japan; 4 Department of Neuropsychiatry, Keio University School of Medicine, 35 Shinanomachi, Sinjuku-ku, Tokyo, 160-8582 Japan; 5 Department of Neuropathology, Graduate School of Medicine, University of Tokyo, 7-3-1 Hongo, Bunkyo-ku, Tokyo, 113-0033 Japan; 6 Division of System Medical Science, Institute for Comprehensive Medical Science, Fujita Health University, 1-98, Dengakugakubo, Kutsukake-cho, Toyoake, Aichi 470-1192 Japan; 7 Central Institute for Experimental Animals (CIEA), 3-25-12 Tonomachi, Kawasaki-ku, Kawasaki, 210-0821 Japan; 8 Division of Pharmacology, Faculty of Pharmacy, Keio University, 1-5-30, Shibakoen, Minato-ku, Tokyo, 105-8512 Japan

**Keywords:** 5xFAD, Alzheimer’s disease, Amyloid β, Aquaporin-4, Epilepsy

## Abstract

Aquaporin-4 (AQP4) has been suggested to be involved in the pathogenesis of neurodegenerative diseases including Alzheimer’s disease (AD), which may be due to the modulation of neuroinflammation or the impairment of interstitial fluid bulk flow system in the central nervous system. Here, we show an age-dependent impairment of several behavioral outcomes in 5xFAD AQP4 null mice. Twenty-four-hour video recordings and computational analyses of their movement revealed that the nighttime motion of AQP4-deficient 5xFAD mice was progressively reduced between 20 and 36 weeks of age, with a sharp deterioration occurring between 30 and 32 weeks. This reduction in nighttime motion was accompanied by motor dysfunction and epileptiform neuronal activities, demonstrated by increased abnormal spikes by electroencephalography. In addition, all AQP4-deficient 5xFAD mice exhibited convulsions at least once during the period of the analysis. Interestingly, despite such obvious phenotypes, parenchymal amyloid β (Aβ) deposition, reactive astrocytosis, and activated microgliosis surrounding amyloid plaques were unchanged in the AQP4-deficient 5xFAD mice relative to 5xFAD mice. Taken together, our data indicate that AQP4 deficiency greatly accelerates an age-dependent deterioration of neuronal function in 5xFAD mice associated with epileptiform neuronal activity without significantly altering Aβ deposition or neuroinflammation in this mouse model. We therefore propose that there exists another pathophysiological phase in AD which follows amyloid plaque deposition and neuroinflammation and is sensitive to AQP4 deficiency.

## Introduction

Alzheimer’s disease (AD) is a progressive neurodegenerative disorder and is the most common cause of dementia. Histopathologically, AD is characterized by senile plaques, extracellular deposits consisting mainly of amyloid β (Aβ) peptide, and neurofibrillary tangles, intraneuronal deposits of hyperphosphorylated tau protein. It is well known that reactive astrocytes and activated microglia accumulate around these amyloid plaques in both AD patients and mouse models of AD, and the subsequent neuroinflammation is thought to be involved in AD pathogenesis [6, 19, 39, 45].

Aquaporin-4 (AQP4) is the most abundant water channel in the central nervous system (CNS), contributing to water and ion homeostasis, and is strongly expressed in the perivascular and subpial end-feet of astrocytes [38], although a lower degree of AQP4 polarization to perivascular astrocytic end-foot membranes in humans compared with mice due to higher AQP4 expression in parenchymal astrocytic membranes in human brains was observed [10]. It has been reported that the expression of AQP4 is upregulated around amyloid plaques in AD patients and mouse models of AD [14, 15, 57–59], but the functional significance of these findings are unclear. Interestingly, the AQP4 deficiency in mice modifies neuroinflammatory responses in various situations. For example, astrocytosis and/or microgliosis caused by traumatic acute brain damage were shown to be diminished in AQP4 knockout (KO) mice [17, 33, 44, 46]. Similarly, neuroinflammation induced by lipopolysaccharide (LPS) or experimental autoimmune encephalomyelitis (EAE) was attenuated in AQP4 KO mice, showing the neuroprotective effect of AQP4 deficiency [31, 32, 34]. In contrast, in models of chronic Parkinson’s disease [50], cryoinjury [46], and focal cerebral ischemia [47] AQP4 deficiency enhanced neuroinflammation, leading to severe neuronal damage. Thus, these findings suggest that AQP4 function might also be implicated in the pathogenesis of AD.

Recently, a CNS interstitial solute clearance system known as the glymphatic system has been proposed [18]. In this system, interstitial metabolites and waste, including Aβ, are transported to perivenous spaces surrounding the large deep veins, accompanied by the convective flow of interstitial fluid (ISF). This is driven by the efflux of cerebrospinal fluid (CSF) from the subarachnoid space into the brain parenchyma through paravascular spaces in the same direction as blood flow. This requires AQP4 function [18, 28, 36], suggesting a role for AQP4 in AD pathogenesis which is not associated with neuroinflammation.

Here, we investigated whether AQP4 is involved in the primary neurodegenerative course of AD, namely the deposition of amyloid plaques and neuroinflammation, or the subsequent events of AD pathogenesis. For this purpose, we utilized 5xFAD mice as an Alzheimer’s model, and 5xFAD mice deficient in AQP4. 5xFAD mice are one of the most widely used models for AD and produce high levels of Aβ_42_ and immediately accumulate amyloid plaques by 2 months of age [40]. Using this model, we found an age-dependent deterioration of neuronal function in 5xFAD mice which is sensitive to AQP4 deficiency and progresses independently of Aβ deposition or neuroinflammation.

## Materials and methods

### Animals

AQP4 knockout mice were generated as described previously [17] (acc. No. CDB0758 K: http://www.cdb.riken.jp/arg/mutant%20mice%20list.html) and maintained by crossing with C57BL/6JJcl (CLEA Japan Inc. Tokyo, Japan). B6.Cg-Tg (APPSwFlLon,PSEN1*M146L*L286V)6799Vas/Mmjax (5xFAD) [40] were obtained from Mutant Mouse Resource & Research Centers. The mice were housed in polycarbonate cages (3–4 animals per cage) at 22–24 °C under a 12 h light /12 h dark cycle with food and water ad libitum. Numbers of mice used in this study are shown in Supplemental Table S1. We detected age-dependent reduction in body weight of 5xFAD mice (Supplemental Fig. S1a), which has also reported previously [23], and we did not see an effect of AQP4 deficiency on the reduction in body weight of 5xFAD aged around 8 month (Supplemental Fig. S1a).

### Reverse transcription quantitative polymerase chain reaction (RT-qPCR)

Total RNA was extracted from cerebral hemispheres with Isogen (Nippon Gene Co., Ltd., Toyama, Japan). First-strand cDNAs were synthesized using SuperScript VILO Master Mix (Thermo Fisher Scientific, Waltham, MA). The qPCR analysis was performed using KOD SYBR qPCR mix (Toyobo) and the Applied Biosystems StepOne Real Time PCR system (Thermo Fisher Scientific). Primers used for the qPCR are listed in Supplemental Table S2.

### Immunohistochemistry

Mouse brain slice sections were prepared as described previously [12] with slight modifications. Paraffin sections with thickness of 5 μm was subjected to immunostaining using a Lica Bond-Max automatic immunostainer (Leica Biosystems, Mount Waverley, VIC, Australia). Used antibodies are rabbit anti-beta Amyloid 1–42 antibody (mOC64, 1:1000, abcam, Cambridge, UK) rabbit anti-AQP4 (1:30000, Atlas Antibodies AB, Bromma, Sweden), rabbit anti-Glial fibrillary acidic protein (GFAP) (1:2000, abcam), and rabbit anti-Iba1 (1:2000, FUJIFILM Wako Pure Chemical Corporation, Osaka, Japan) antibodies. The number and size of plaques were analyzed using ImageJ ver. 2.

### Clearing brains

We used tissue clearing protocol based on a protocol for Clear, Unobstructed Brain/Body Imaging Cocktails and Computational analysis (CUBIC) [29, 51]. Brains were removed from mice anaesthetized by inhalation administration of isoflurane followed by perfusion fixation with 4% paraformaldehyde (PFA). The brains were immersed in 4% PFA at 4 °C for 2 h and were cut sagittally along the midline. The cerebral hemispheres were further immersed in 4% PFA at 4 °C overnight and transferred to 50% CUBIC-L solution [51] to incubate at 37 °C for 6 h with gently shaking. Then the hemispheres were transferred to CUBIC-L [51] to incubate at 37 °C with gently shaking for 7 days, changing the solution every 2 days. For immunofluorescent staining, the hemispheres were washed with TBS-T for 1 h followed by washed twice with TBS containing 0.01% sodium azide for 2 h. Then the hemispheres were stained with Alexa Fluor 488-labeled monoclonal anti-β amyloid, 1–16 (6E10, 1:200, BioLegend, San Diego, CA) and Amylo-Glo RTD Amyloid Plaque Stain Reagent (1:100, Biosensis, Thebarton, Australia) in TBS containing 0.01% sodium azide, 0.5% Triton X-100, and 2.5% BSA at room temperature for 3 days. The stained hemisphares were washed three times with TBS containing 0.01% sodium azide and transferred to Sca*l*eCUBIC-2 [29] to incubate at 37 °C for 2 days with gentle shaking. The cleared brains in 80% Sca*l*eCUBIC-2 were observed with Lightsheet Z.1 (Carl Zeiss, Oberkochen, Germany). Obtained images were stitched with arivis Vision4D ver. 2.12.3 (arivis AG, Munich, Germany), and analyzed with Imaris ver. 9.1.2 (Biplane AG, Zurich. Switzerland).

One-hundred-μm sections were also cleared using the CUBIC method and stained with Amylo-Glo RTD Amyloid Plaque Stain Reagent, Cy3-conjugated monoclonal anti-GFAP (G-A-5, 1:200, Sigma-Aldrich corp. St. Louis, MO), Red Fluorochrome(635)-conjugated rabbit anti-Iba1 (1:200, FUJIFILM Wako Pure Chemical Corporation), and rabbit anti-AQP4 (1:200, Sigma-Aldrich corp.) followed by Alexa Fluor 488-labeled goat anti-rabbit IgG (1:200, Thermo Fisher Scientific). The sections were mounted on a slide using ProLong™ Glass Antifade Mountant (Thermo Fisher Scientific) and observed with LSM 710 laser scanning confocal microscope (Carl Zeiss).

### Digital Vivarium

Vium Digital Smart Houses consist of standard individually ventilated cage slotted in Vium’s proprietary rack system. The Digital Vivarium test was described previously [35]. Briefly, Vium Digital Smart Houses are outfitted with sensors and a high-definition camera that enable continuous monitoring of animals. The Vium Digital Platform obtains and maintains a digital record of the data analytics on motion. This study used the validated Vium Motion (m/sec). Daytime motion (collected from 0600 to 1800 PDT), nighttime motion (collected from 1800 to 0600 PDT).

### Measurement of Aβ

Extraction of soluble and insoluble Aβ was performed as described in Hashimoto et al. [13]. TBS and 70% formic acid fractions were used as soluble and insoluble Aβ fractions, respectively. Concentration of Aβ_40_ and Aβ_42_ was determined using Human/Rat βAmyloid(40) ELISA Kit Wako II and Human/Rat βAmyloid(42) ELISA Kit Wako (FUJIFILM Wako Pure Chemical Corporation, Osaka, Japan), respectively.

For in vivo measuring Aβ concentration in interstitial fluid, mice were anesthetized with isoflurane. A guide cannula (an outer diameter of 0.72 mm and an inner diameter of 0.64 mm; Eicom, Kyoto, Japan) was stereotactically inserted in the right hippocampus (AP: − 3.1 mm and LT: 2.5 mm from bregma, and V: 1.3 mm) [43] and cemented. A dummy cannula (Eicom) was placed in the guide cannula. After 3–5 days of recovery period, mice were subjected to microdialysis under the freely moving condition (AtmosLM system, Eicom). A 2-mm microdialysis probe (PEP-4-02, 2 mm, 1000 kDa molecular weight cut-off, Eicom) was inserted in the guide cannula and perfusion buffer (147 mM NaCl, 4 mM KCl, 2.3 mM CaCl_2_, 0.15% bovine serum albumin, filtrated with 0.2 μm syringe filter) was circulated by peristaltic pump (ERP-10, Eicom) and microsyringe pump (ESP-64, Eicom) at a flow rate of 10 μl/min for 3 h followed by 1 μl/min for 1 h. After 4 h of perfusion, samples were collected at 2 h/fraction in a fraction collector (EFC-96, Eicom) refrigerated at 4 °C. Collected samples were stored at − 80 °C until ELISA assay.

### Electroencephalography

The animals were deeply anesthetized with a mixture of ketamine and xylazine (100 mg/kg and 10 mg/kg, respectively, i.p.) in accordance with guideline from Japanese Association for Laboratory Animal Medicine (JALAM), and then fixed to a stereotaxic apparatus (SM-15, Narishige Scientific Instrument, Tokyo, Japan). Their body temperature was maintained at 37 ± 0.5 °C using a heating pad (FHC-MO, Muromachi Kikai, Tokyo, Japan) during the surgical procedure. A longitudinal incision was made and the skull surface was exposed. The periosteum and blood were removed thoroughly. Two craniotomies with a diameter of 1 mm were prepared using a drill. The coordinates of the craniotomies were 0.5 mm posterior to Bregma, 2.5 mm lateral from the midline to the left for the somatosensory cortex, and 7.5 mm posterior to Bregma, 0.3 mm lateral from the midline to the left for the cerebellum [43]. In the individual craniotomies, Teflon-coated silver wire electrodes (786,500, A-M Systems, WA, USA) were implanted above the brain surface as an EEG electrode and a reference. The exposed skull was then covered with a dental cement (Super-Bond C&B, Sun Medical, Shiga, Japan). After completing surgical procedure, the mouse was returned to its homecage for recovery.

After recovery for approximately 1 week, EEG recording was performed. EEG signals were transferred through flexible cables (AWG32, Mogami Cable, Ca, USA) and amplified by 1000-fold, bandpass filtered between 1 and 1000 Hz (Model 3000, A-M Systems, WA, USA), and digitized at 1000 Hz using an analog to digital converter (cDAQ-9178, National Instruments, TX, USA).

EEG data were analyzed using custom-written programs in MATLAB (2017a, MathWorks, MA, USA). Epileptiform spikes were defined as sharp (< 50 ms) negative deflections with amplitudes exceeding twice the baseline EEG [37]. Number of the epileptiform large EEG waveform in each mouse were counted in all recording sessions.

### Grip strength test

The forelimb grip strength was measured using grip strength meter (O’Hara & Co., Tokyo, Japan). The mice were lifted by their tails so that their forepaws could grasp a wire grid of the apparatus. The mice were gently pulled backward by the tail until they released the grip. The peak force was recorded in Newton (N).

### T-maze test

Spontaneous alternation task was conducted to assess working memory, as previously described [48]. The mice were subjected to a session consisting of 10 trials. Each trial consisted of a forced choice followed by a free choice. In the forced-choice trial, the mice were forced to enter either the left or right arm of the T-shaped platform of the maze. After the 10-s period, the mouse could return to the starting compartment, and a free-choice trial was started. In the free-choice trial, the mice were allowed to choose one of the arms. The percentage of correct responses in which the mice entered the arm opposite to their choice in the forced-choice trial during the free-choice trial was calculated. Total distance traveled and latency were also measured. Data acquisition and analysis were performed automatically using ImageTM software, based on the public domain ImageJ program (http://rsb.info.nih.gov/ij/), was developed and modified by Tsuyoshi Miyakawa.

### Statistical analysis

For Fig. 2, statistical analysis was performed using SPSS ver. 25 (IBM, Armonk, NY, USA). Data were analyzed using one-way ANOVA, followed by the Bonferroni method. For Figs. 3, 4, 5a, b, and 6; and Supplemental Fig. S5 statistical analysis was performed using JMP ver. 14.0.0 (SAS Institute Inc., Cary, NC, USA). Data were analyzed using two-sided student t-test for Figs. 4, 5a, and b; and one-way ANOVA followed by the Tukey-Kramer method for Figs. 3 and 6 and Supplemental Fig. S5. For Fig. 5c, d, e, and f, statistical analysis was conducted using Prism5 (GraphPad Software Inc., San Diego, CA, USA). Data were analyzed using two-way ANOVA. For Supplemental Fig. S1, statistical analysis was conducted using StatView (SAS Institute Inc.). Data were analyzed using one-way ANOVA (a, b), two-way repeated measures ANOVA (c, d).

## Results

### AQP4 deficiency accelerated a loss of activity and induced motor dysfunction in female 5xFAD mice

We observed that female 5xFAD/AQP4 KO mice exhibited motor dysfunction, for example demonstrating difficulty in holding food while eating. This phenotype started around 32 weeks of age, and the mice then gradually became immobilized. Males also showed the same phenotype which appeared much later as compared with females. The motor dysfunction was confirmed by an age-dependent loss of grip strength (Supplemental Fig. S1b).

**Fig. 1:**
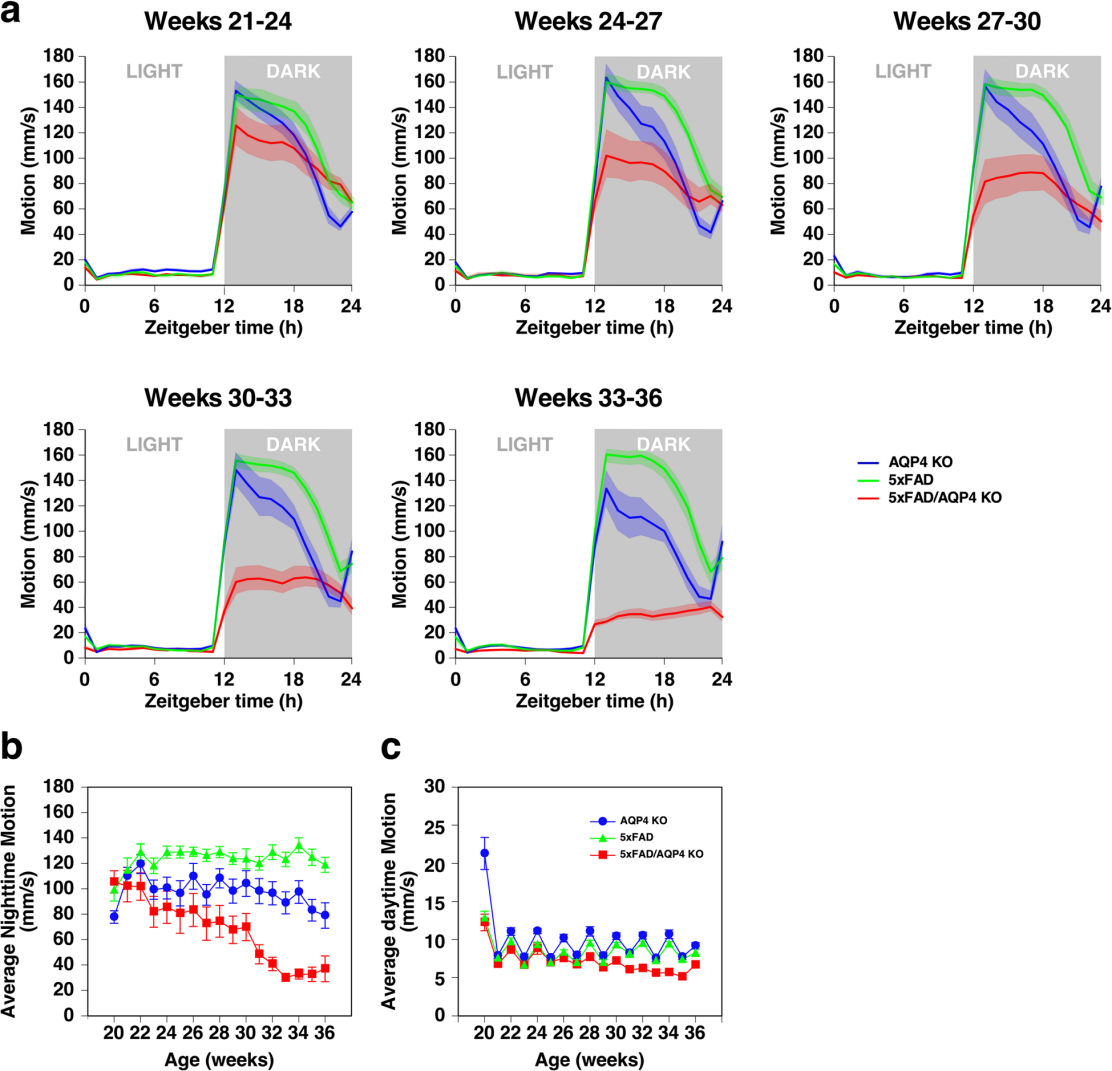
Effect of AQP4 deficiency on the activity of 5xFAD female mice. **a** Average circadian motion of 5xFAD (green), AQP4 KO (blue), and 5xFAD/AQP4 KO (red) mice. **b** and **c** Average nighttime (**b**) and daytime (**c**) motions of 5xFAD (green triangles), AQP4 KO (blue circles), and 5xFAD/AQP4 KO (red squares) mice. Mice became active during the daytime after cage change occurred every 2 weeks. Values are mean ± S.E.M of 11 individuals

To clarify the onset and quantify the degree of this immobility, we observed the behavior of female 5xFAD, AQP4 KO, and 5xFAD/AQP4 KO mice using Digital Vivarium™ (Vium, San Mateo, CA), a 24-h video recording and computational analysis system which assesses the movements of individual mice, between 20 and 36 weeks of age. We used female mice since male mice hardly showed behavioral abnormalities during this period, and females exhibited more severe amyloidosis [40]. During the initial 3 weeks of the analysis, all three groups exhibited normal circadian rhythm (Fig. 1a). The system clearly detected an age-dependent reduction in nighttime activity in the 5xFAD/AQP4 KO mice (Fig. 1a, red lines). Average nighttime motion gradually reduced with age, and sharply deteriorated during 30 to 32 weeks of age (Fig. 1b), with the mice exhibiting movement less than 50 mm/sec. The average age of onset of movement less than 50 mm/sec was 28.5 ± 1.3 weeks, with a range of 23 to 34 weeks. In contrast, the 5xFAD mice were constantly active during the night throughout the four-month analysis period, even compared with the AQP4 KO mice (Fig. 1a and b, green and blue lines). The analysis system detected that daytime motion in the 5xFAD/AQP4 KO mice was also reduced starting at approximately 28 weeks of age (Fig. 1c).

### Epileptiform activity in 5xFAD mice

**Fig. 2:**
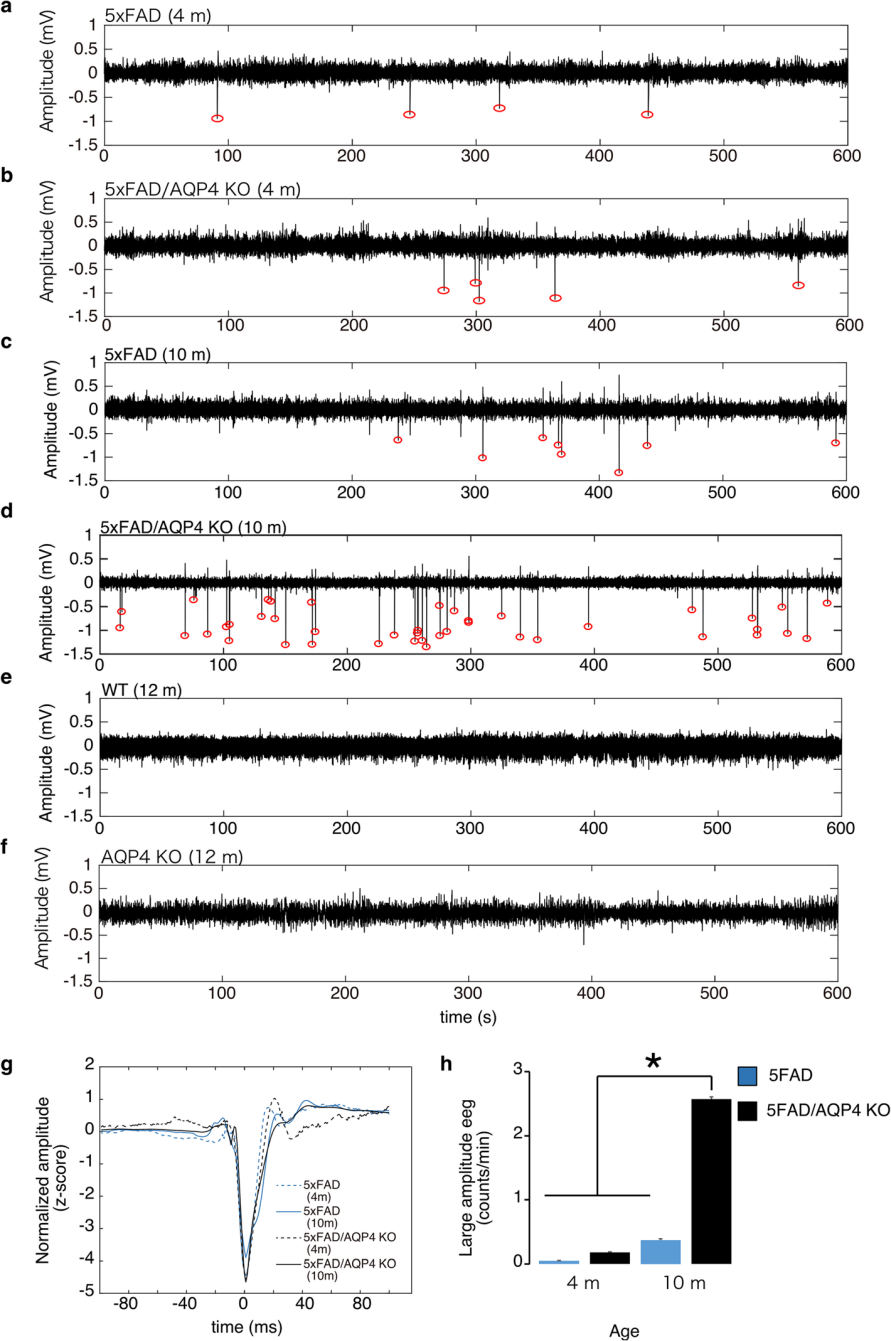
Electroencephalography of 5xFAD female mice. **a-f** Representative electroencephalographic (EEG) traces from 4-month-old 5xFAD (**a**), 4-month-old 5xFAD/AQP4 KO (**b**), 10-month-old 5xFAD (**c**), 10-month-old 5xFAD/AQP4 KO (**d**), 12-month-old wild-type (**e**), and 12-month-old AQP4 KO (**f**) mice. Red circles indicate epileptiform activity. **g** Mean traces of epileptiform spike waveforms from 5xFAD (4 months), 5xFAD/AQP4 KO (4 months), 5xFAD (10 months), and 5xFAD/AQP4 KO (10 months) mice. **h** Mean epileptiform spike counts per minute were obtained from 5xFAD (4 months, *n* = 5), 5xFAD/AQP4 KO (4 months, *n* = 5), 5xFAD (10 months, *n* = 6), and 5xFAD/AQP4 KO (10 months, *n* = 10) mice. Values are mean ± S.E.M. * (*P* < 0.01) represents significant differences of 10-month-old 5xFAD/AQP4 KO versus each of the other groups


We found that all 11 5xFAD/AQP4 KO mice exhibited convulsions (Supplemental Movie S1) at least once after the onset of loss of nighttime activity, detected using Digital Vivarium™. Since multiple AD model animals are known to exhibit epileptiform activity at early stages [2], we hypothesized that 5xFAD mice also have a predisposition to produce epileptiform activity and AQP4 deficiency exacerbates this phenotype. We recorded electroencephalograms (EEG) of the 5xFAD mice at 4 months of age, when Aβ deposition, reactive astrocytosis and activated microgliosis are prominent [40]. In this early stage of the disease, epileptiform spikes were detected, though their frequency was low and no significant difference was noted between the 5xFAD and 5xFAD/AQP4 KO groups (Fig. 2a, b, g and h). However, at 10 months of age, these epileptiform spikes dramatically increased in the 5xFAD/AQP4 KO mice compared with the 5xFAD mice of the same age (Fig. 2c and d). On a number of occasions, we observed convulsions during the EEG recordings (Supplemental Fig. S2 and Supplemental Movie S2), implying that the increase in epileptiform activity results in convulsions. Aged wild-type and AQP4-KO mice (12 months) did not exhibit any epileptiform activity (Fig. 2e and f). Taken together, this suggests that 5xFAD mice have a predisposition to convulsions and AQP4 deficiency facilitates the onset of convulsions.

**Additional file 1.** Supplemental Movie S1.
**Additional file 2.** Supplemental Movie S2.


### Age-dependent increase in AQP4 expression around amyloid plaques in 5xFAD mice

**Fig. 3:**
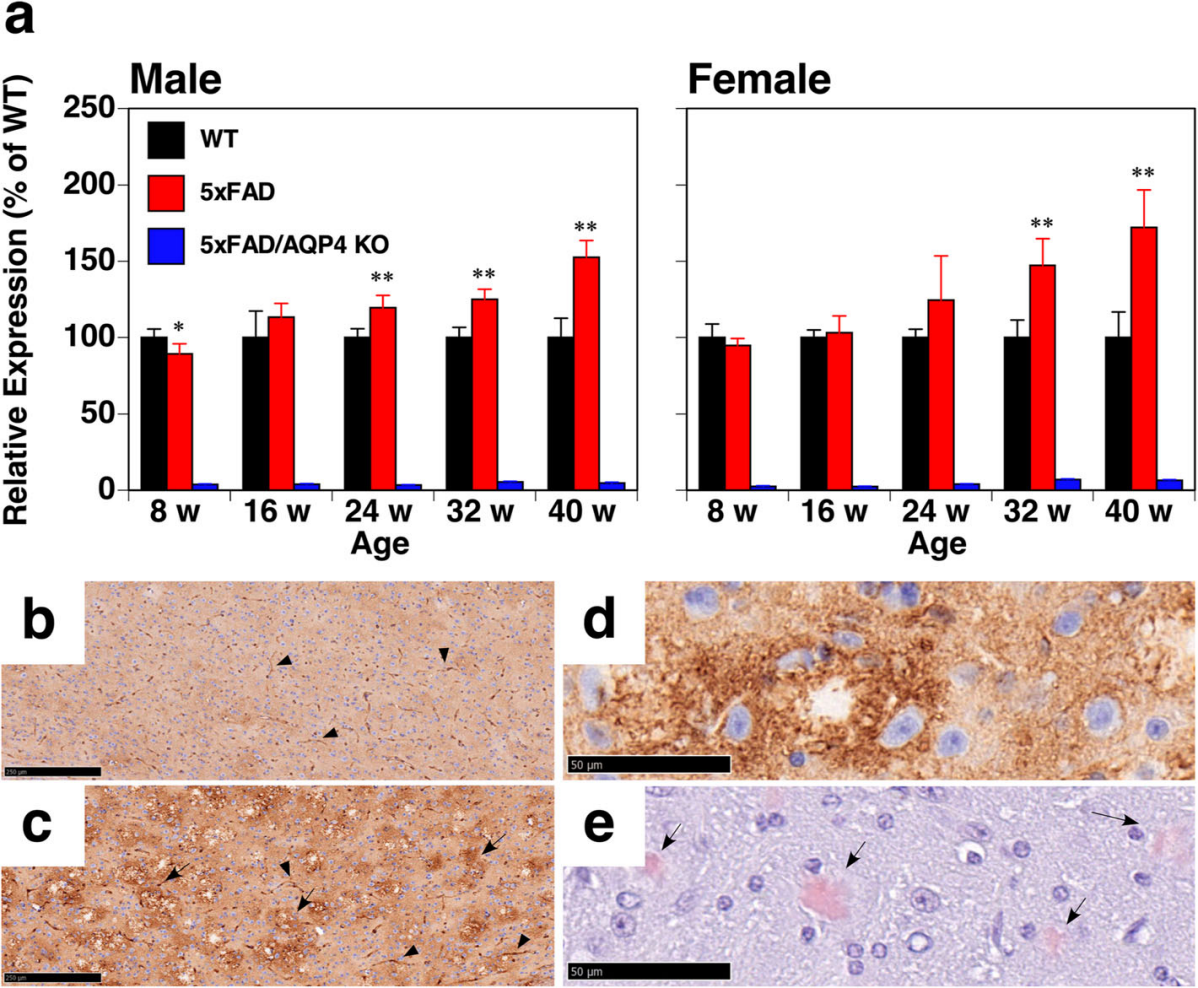
Expression of AQP4 in 5xFAD mice. **a** qPCR analysis of brain hemispheres from wild-type (black columns), 5xFAD (red columns), and 5xFAD/AQP4 KO (blue columns) mice. Values are mean ± S.E.M of 5–6 individuals. *(*P* < 0.01) and **(*P* < 0.001) represent significant differences versus wild-type mice. **b** and **c** Comparison of AQP4 expression in the cortices of wild-type (**b**) and 5xFAD (**c**) mice. Perivascular staining of AQP4 is denoted by arrow heads. The expression of AQP4 around unstained spots is denoted by arrows. **d** and **e** Aberrant expression of AQP4 (**d**) was observed around amyloid plaques stained with Congo red (**e**). Scale bars = 250 μm (**b** and **c**) and 50 μm (**d** and **e**)

To understand the role of AQP4 in AD pathology, especially after the accumulation of Aβ in the brain parenchyma, we next examined the expression of AQP4 in the 5xFAD mice. As shown in Fig. 3a, AQP4 transcription was upregulated at 24 weeks of age in both male and female 5xFAD mice. The level of the AQP4 transcript increased in an age-dependent manner, mirroring the age-dependent progression of immobility observed in AQP4 deficient 5xFAD mice. The AQP4 protein accumulated in a variety of brain regions, including the cortex, hippocampus, thalamus, and brain stem of 40-week-old female 5xFAD mice (Supplemental Fig. S3a-c). Perivascular localization of AQP4 was maintained at 40 weeks of age (Fig. 3b and c, arrow heads). Aberrant expression of AQP4 was also observed around unstained regions in the 5xFAD mouse brain (Fig. 3c, arrows), which were determined to be amyloid plaques via Congo red staining (Fig. 3d and e) as well as 100-μm sections stained with AmyloGlo and anti-AQP4 antibody (Supplemental Fig. S4 and Supplemental Movies S3 and S4) [14, 15, 57–59].

### AQP4 deficiency did not significantly affect amyloid deposition or neuroinflammation in 5xFAD mice

**Fig. 4:**
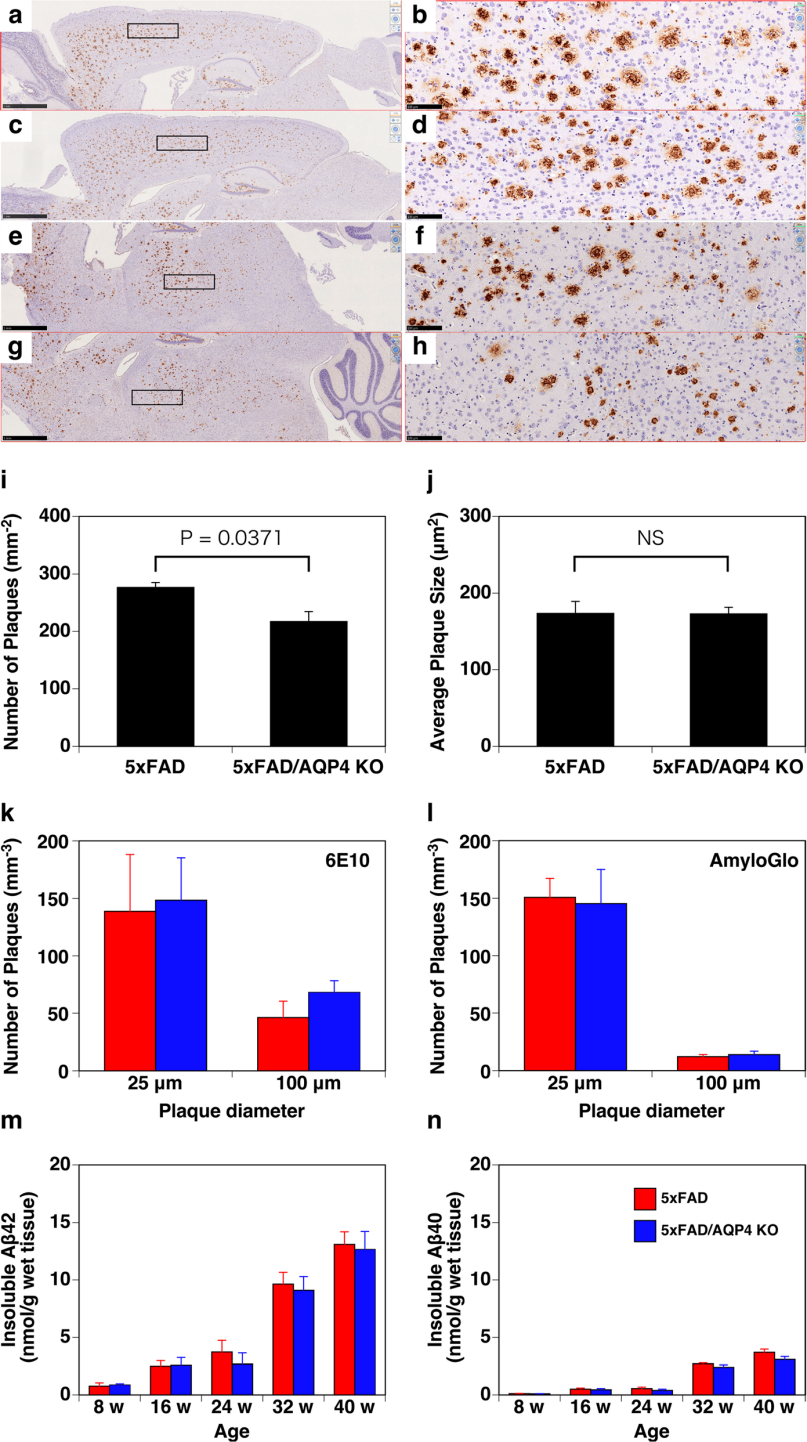
Effect of AQP4 deficiency on the deposition of Aβ in parenchyma of 5xFAD female mice. **a-h** Representative immunohistochemical images of sagittal brain sections of 40-week-old 5xFAD/AQP4 KO (**a**, **b**, **e**, and **f)** and 5xFAD (**c**, **d**, **g**, and **h**) mice stained with an anti-Aβ_42_ antibody. **b**, **d**, **f**, and **h** are magnified images of **a**, **c**, **e**, and **g**, respectively, indicated by boxes. Scale bars = 1 mm (**a**, **c**, **e**, and **g**) and 100 μm (**b**, **d**, **f**, and **h**). **i** and **j** Quantification of the number of amyloid plaques and plaque size in the cortical region. The average number of plaques (**i**) and plaque size (**j**) in ten fields in two sections of cortical regions from each animal (*n* = 3), including (**a**) and (**c**), were counted using ImageJ. Values are mean ± S.E.M of 3 individuals. **k** and **l** Quantification of the number of amyloid plaques of approximately 25 and 100 μm in diameter calculated by Imaris in the 3D imaging of cleared hemispheres from 45-week-old 5xFAD (red columns, Supplemental Movie S5) and 5xFAD/AQP4 KO (blue columns, Supplemental Movie S6) mice stained with Alexa Fluor 488-labeled 6E10 to identify total Aβ (**k,** green in movies) and Amylo-Glo to identify Aβ fibrils (**l**, magenta in movies). Values are mean ± S.E.M of 3 individuals. **m** and **n** The amounts of insoluble Aβ_42_ (**m**) and Aβ_40_ (**n**) extracted from the cerebral hemispheres of 5xFAD (red columns) and 5xFAD/AQP4 KO (blue columns) mice were determined by ELISA. Values are mean ± S.E.M of 6–7 individuals

To explore the mechanisms by which AQP4 deficiency promotes motor dysfunction in 5xFAD mice, we then stained brain sections with an anti-Aβ_42_ antibody. As shown in Fig. 4, similar plaque accumulation was observed in the 5xFAD and 5xFAD/AQP4 KO mice (Fig. 4a-h). We did not observe perivascular deposition of Aβ in the 5xFAD mice, regardless of the expression of AQP4 (Fig. 4a-h). The number of plaques in the cortical region was slightly greater in the 5xFAD mice (Fig. 4i). The average size of plaques was also determined; however, there was no significant difference between 5xFAD and 5xFAD/AQP4 KO mice although the 5xFAD/AQP4 KO mice seemed to accumulate some large plaques with areas more than 2.2 × 10^3^ μm^2^ (Fig. 4a, b, e, and f compared to c, d, g, and h; Supplemental Fig. S5), which is consistent with a previous report [49]. Similar results were obtained in the 3D imaging of cleared hemispheres from 45-week-old female 5xFAD (Supplemental Movie S5) and 5xFAD/AQP4 KO (Supplemental Movie S6) mice, using the CUBIC method and anti-Aβ (Fig. 4k; Supplemental Movies S5 and S6, green) and Amylo-Glo (Fig. 4l; Supplemental Movies S5 and S6, magenta) staining followed by analysis using Imaris.

We measured insoluble Aβ by ELISA up to 40 weeks of age and found that the 5xFAD mice accumulated insoluble Aβ_42_ with age (Fig. 4m). Aβ_40_ was also detected in the insoluble fraction, but the level of Aβ_40_ was ~ 30% of that of Aβ_42_ (Fig. 4n). Consistent with the results obtained by histological analysis, there was no significant difference in insoluble Aβ accumulation between the 5xFAD and 5xFAD/AQP4 KO mice. The expression of neprilysin was reduced in the 5xFAD mice as previously reported [9, 16], but was not affected by the loss of AQP4 (Supplemental Fig. S6). Other enzymes responsible for Aβ production and degradation were not altered in the 5xFAD mice, regardless of AQP4 expression, as determined by qPCR analysis (Supplemental Fig. S6). Taken together, little effect of AQP4 deficiency on Aβ deposition in the parenchyma was observed in the 5xFAD mice.

**Fig. 5:**
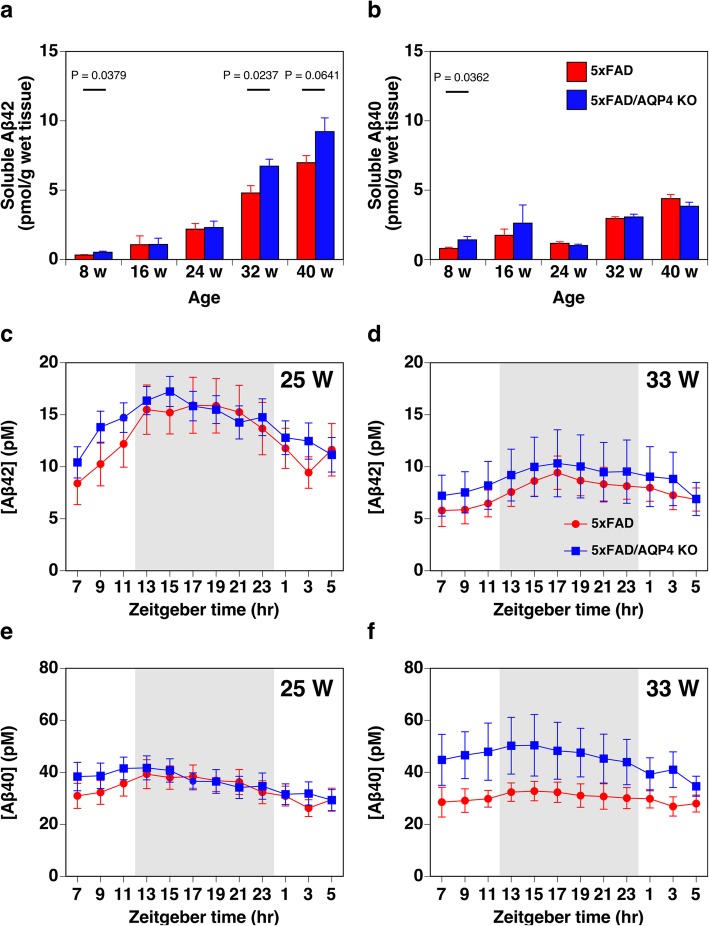
Effect of AQP4 deficiency on soluble Aβ levels in 5xFAD female mice. **a** and **b** The amounts of soluble Aβ_42_ (**a**) and Aβ_40_ (**b**) extracted from the cerebral hemispheres of 5xFAD (red columns) and 5xFAD/AQP4 KO (blue columns) mice were determined by ELISA. One individual 32-week-old 5xFAD female mouse showed more than 4.0 and 7.5 times higher soluble Aβ_42_ and Aβ_40_ concentrations, respectively, compared with the average of the other seven individuals of the same group. Therefore, this mouse was excluded from all analyses, including insoluble Aβ level shown in Fig. 4m and n. Values are mean ± S.E.M of 6–7 individuals. **c**-**f** Time course of ISF-soluble Aβ_42_ (**c** and **d**) and Aβ_40_ (**e** and **f**) concentrations in hippocampal region of 5xFAD (red circles) and 5xFAD/AQP4 KO (blue squares) females at 25 weeks (**c** and **e**) and 33 weeks (**d** and **f**) of ages measured by ELISA of samples obtained by microdialysis. Values are mean ± S.E.M of 6–9 individuals

In addition to insoluble Aβ, we also measured soluble Aβ using ELISA. Total soluble Aβ_42_ gradually increased in an age-dependent manner, and the level of soluble Aβ_42_ was higher in the 5xFAD/AQP4 KO mice than the 5xFAD mice between 32 and 40 weeks of age, although the difference was not statistically significant at 40 weeks of age (Fig. 5a). Since Cirrito et al. [7] suggested that soluble Aβ in tissue lysates contains not only interstitial soluble Aβ but also Aβ loosely associated to amyloid plaques solubilized after tissue lysis, even in mild buffer, and thus it is not truly soluble and diffusible in the extracellular space. Therefore, we also measured interstitial soluble Aβ in vivo using microdialysis followed by ELISA. Similarly to the soluble Aβ in tissue lysates, interstitial soluble Aβ_42_ in vivo in females at 33 weeks of age, but not at 25 weeks of age, tended to be higher in the 5xFAD/AQP4 KO mice than in age-matched 5xFAD mice (Fig. 5c and d). Total soluble Aβ_40_ predominantly accumulated up to 16 weeks of age, and then did not drastically increase up to 40 weeks of age regardless of AQP4 expression (Fig. 5b). The interstitial soluble Aβ_40_ measured in vivo in females at not 25 weeks but 33 weeks of age tended to be higher in the 5xFAD/AQP4 KO mice as seen in Aβ_42_ (Fig. 5e and f). Note that the levels of soluble Aβ_42_ and Aβ_40_ were high in the 5xFAD/AQP4 KO mice as early as 8 weeks of age, when amyloid plaques are barely detectable (Fig. 5a and b).

**Fig. 6:**
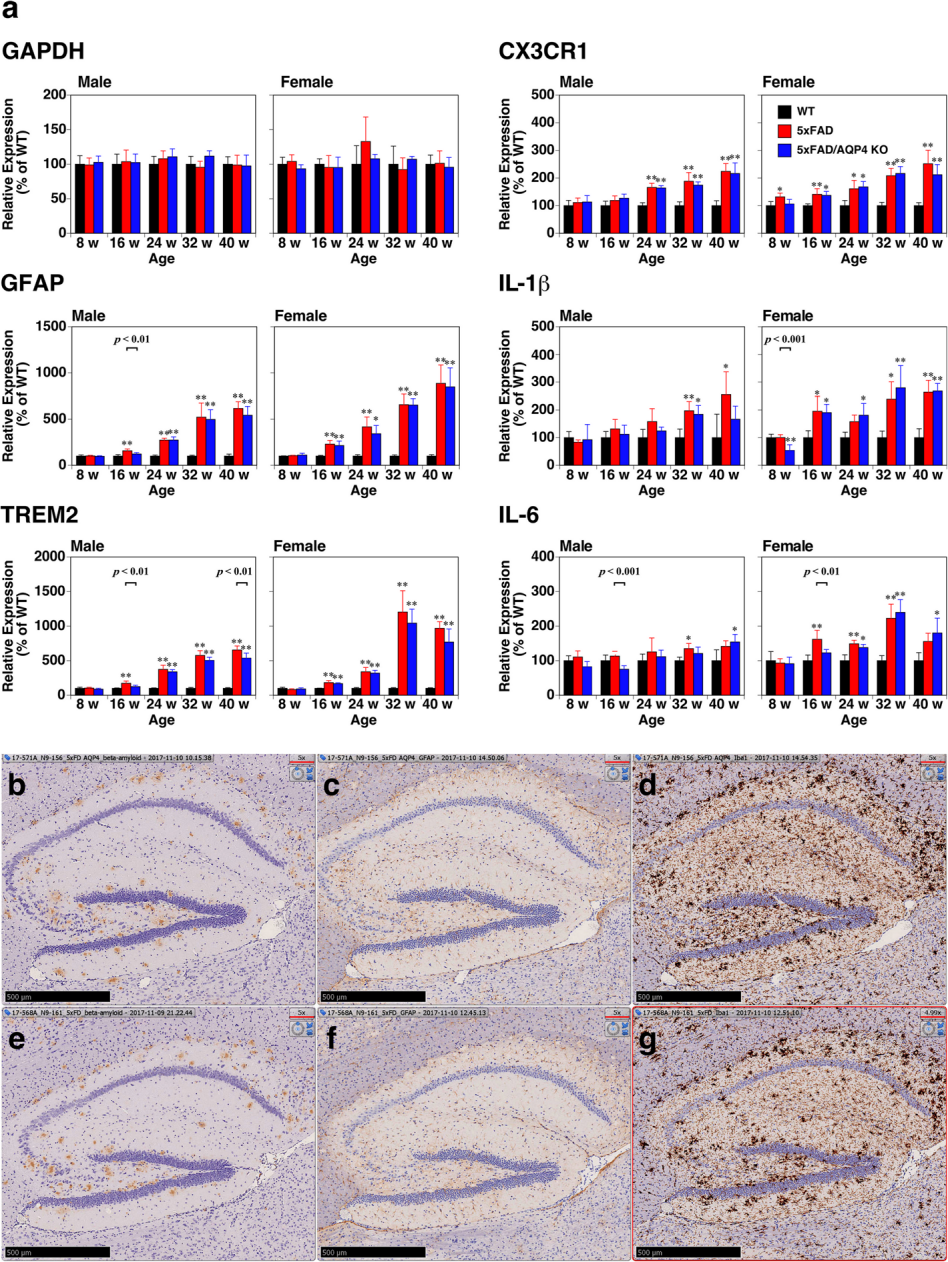
Effect of AQP4 deficiency on neuroinflammatory responses in 5xFAD mice. **a** qPCR analysis of brain hemispheres from wild-type (black columns), 5xFAD (red columns), and 5xFAD/AQP4 KO (blue columns) mice. The level of the transcript of each gene in 5xFAD and 5xFAD/AQP4 KO mice was determined as the % of that of age-matched wild-type mice. Values are mean ± S.E.M of 5–6 individuals. *(*P* < 0.01) and **(*P* < 0.001) represent significant differences versus wild-type mice. The primers used are listed in Supplemental Table S2. **b**-**g** Immunostaining of Aβ (**b** and **e)**, GFAP (**c** and **f**), and Iba1 (**d** and **g**) in the hippocampal region of 5xFAD/AQP4 KO (**b**-**d**) and 5xFAD (**e**-**g**) mice. Scale bars = 500 μm

We then examined whether the loss of AQP4 affected glial responses against amyloid deposition by quantifying the transcripts of some markers of gliosis by qPCR. GFAP, a marker of reactive astrocytes, was significantly increased at 16 weeks of age and continuously increased with age both in the 5xFAD and 5xFAD/AQP4 KO mice, though there was no statistically significant difference between the groups (Fig. 6a). A homeostatic microglial marker, CX3C chemokine receptor 1 (CX3CR1) [26, 27], also increased with age in these mice but did not dramatically increase (Fig. 6a). This was also the case with neuroinflammatory markers such as IL1β and IL6 (Fig. 6a). In contrast, the expression of triggering receptor expressed on myeloid cell 2 (TREM2), an inducer of the microglial neurodegenerative phenotype [26, 27], dramatically increased between 24 and 32 weeks of age, though again there was no significant difference in microglial response between the 5xFAD and 5xFAD/AQP4 KO mice (Fig. 6a). Histological analysis also revealed little effect of AQP4 deficiency on gliosis in the 5xFAD mice (Fig. 6b-g). We also checked the morphology of astrocytes and microglia near amyloid plaques in cortical region of 5xFAD mice using cleared 100-μm sections. As far as we have observed after anti-GFAP and anti-Iba1 staining, there were no obvious differences in their morphology between 5xFAD and 5xFAD/AQP4 KO mice (Supplemental Fig. S7 and Supplemental Movies S7, S8, S9, S10). These observations support the idea that depleting AQP4 function does not affect gliosis or neuroinflammatory responses in 5xFAD mice. In agreement with these results, we did not observe a difference in memory deficit between the 5xFAD and 5xFAD/AQP4 KO mice at 26–30 weeks of age, determined using a T-maze test (Supplemental Fig. S1c and d).

## Discussion

In the present study, we did see behavioral abnormality resulting from AQP4 deficiency, namely a reduction in nighttime activity in 5xFAD/AQP4 KO mice (Fig. 1a). This was not a direct effect of AQP4 deficiency because the symptom was not observed in AQP4 KO mice at any point during their life span. It should be noted that, although we did not detect any reduction in nighttime activity in 5xFAD mice during our analysis using Digital Vivarium™, we did detect a similar loss of activity in 5xFAD mice much later in their life span. This was consistent with previous reports [23, 41], indicating that AQP4 deficiency greatly accelerates the reduction of nighttime activity in 5xFAD mice. This behavioral abnormality is a phenomenon that occurred independently of amyloid plaque formation in the parenchyma of 5xFAD mice for the following reasons. We observed little difference between the 5xFAD and 5xFAD/AQP4 KO mice in the number of amyloid plaques in the cerebral cortex as determined by immunohistochemistry and in cleared hemispheres (Fig. 4k-l). In addition, we observed age-dependent accumulation of insoluble Aβ; however, there was no significant effect of AQP4 deficiency up to 40 weeks of age (Fig. 4m-n). These observations indicated that in 5xFAD mice, AQP4 function is dispensable with regards to the accumulation of amyloid plaques in parenchyma. Furthermore, we did not see any differences between 5xFAD and 5xFAD/AQP4 KO mice in the age-dependent increase of gliosis or subsequent neuroinflammatory response (Fig. 6). This result strongly suggests that not only the accumulation of amyloid plaques, but also glial responses, which may be the direct response of astrocytes and microglia against the amyloid plaques and which include neuroinflammation, are not directly implicated in the reduction in nighttime activity in AQP4-deficient 5xFAD mice.

Recently, Xu et al. have demonstrated the role of AQP4 in Aβ clearance in APP/PS1 mice [57]. Deleting AQP4 from APP/PS1 [21] mice resulted in increased accumulation of amyloid both as plaques in parenchyma and as cerebral amyloid angiopathy (CAA) around blood vessels, as well as increased soluble Aβ levels [57]. These results indicate that AQP4 is implicated in Aβ clearance, probably via the interstitial fluid bulk-flow clearance system [52] in APP/PS1 mice. In contrast, as described above, we did not see any difference in amyloid plaques/insoluble Aβ accumulation between 5xFAD and 5xFAD/AQP4 KO mice. This dissimilarity is attributable to the difference in contribution of interstitial fluid bulk-flow clearance [52] to Aβ clearance from parenchyma between APP/PS1 and 5xFAD mice. In APP/PS1 mice, production of Aβ_40_ is relatively high and formation of amyloid plaques is relatively slow (as early as 6 months of age as compared with as early as 2 months of age in 5xFAD) [11, 22]. In addition, age-dependent CAA was observed in APP/PS1 mice by 6 months of age [11]. Therefore, interstitial fluid bulk-flow likely has a significant role in Aβ clearance [5, 54] in APP/PS1 mice. However, in 5xFAD mice, amyloid plaques appear at as early as 2 months of age [40]. These plaques continuously incorporate Aβ due to the production of high levels of Aβ_42_ [20], likely in cooperation with reduced neprilysin expression (Supplemental Fig. S6) [9, 16]. Importantly, CAA never occurs in 5xFAD mice [56], and consistent with this report, we did not observe any accumulation of Aβ around blood vessels in the 5xFAD mice up to 40 weeks of age, regardless of AQP4 expression (Fig. 4a-h and Supplemental Movies S5 and S6). These observations strongly suggest that the interstitial fluid bulk-flow clearance system hardly contributes to Aβ clearance in 5xFAD mice. Considering the difference between the two models as well as our observations, we conclude that AQP4 function is dispensable in the age-dependent accumulation of amyloid plaques under the conditions in which the interstitial fluid bulk-flow clearance system fails to work for Aβ clearance. This also means that interstitial fluid bulk-flow is the only mechanism for Aβ clearance in which AQP4 is involved. Thus, using 5xFAD mice, we were able to assess the implications of AQP4 after the accumulation of amyloid plaques in the parenchyma.

Strikingly, our 24-h observation of the behavior of individual mice between 20 and 36 weeks of age using Digital Vivarium™ revealed that all 11 5xFAD/AQP4 KO mice experienced convulsions at least once. These convulsions were observed only after the reduction in nighttime movement had occurred (Fig. 1). The comorbidity of AD and neuronal hyperactivity or seizures has been suggested in previous studies on AD patients and transgenic AD mouse models [2]. Other AD models also exhibit convulsions [24, 30, 55] and spontaneous epileptiform discharges [37, 42]. Multiple reports have demonstrated that spontaneous epileptiform activity [1] and a susceptibility to pharmacologically induced seizures [1, 8, 25, 55] were detected before plaque deposition or memory deficits. Therefore, the formation of amyloid plaques does not seem to be a trigger for inducing epileptiform activity. Given previous studies, the epileptiform discharges observed in the early stages in 5xFAD may be an effect of the overexpression of human amyloid precursor protein (APP) from the transgene [3, 4]. Our results, however, strongly suggest that there is a link between the aggravation of epileptiform neuronal activity/convulsions and the observed changes in behavior after 28 weeks of age. It seems likely that factors other than the overexpression of human APP are involved in these symptoms and that a loss of AQP4 critically deteriorates these processes. Previous studies have demonstrated an increased risk of seizures in AD patients [2]. In addition, EEG abnormalities have also been observed in non-epileptic AD patients [2]. Since the onset of seizures follows cognitive impairment, it is suggested that seizures are consequences of AD pathology. In contrast, Vossel et al. suggested that epileptiform activity may contribute to the development of cognitive decline in AD patients and is not just a marker of the end stage of disease [2, 53]. The 5xFAD/AQP4 KO mice model can be a good tool for identifying the relationship between AD and epileptiform activity. For example, it can be used to examine epileptiform activity modulation by antiepileptic drugs and their effect on the accumulation of amyloid plaque, subsequent neuroinflammation, and memory deficit.

It is still unclear why the effects of AQP4 deficiency first appear as late as 28 weeks of age in this model. One possible reason is that the ability of amyloid plaques in the parenchyma to incorporate soluble Aβ is not saturated until around 28 weeks of age in 5xFAD mice. No matter how much AQP4 is implicated in the interstitial fluid bulk-flow clearance system [52], AQP4 function would not be required for Aβ clearance until this parenchymal storage system was saturated because it is highly likely that the interstitial fluid bulk-flow clearance system [52] contributes little to Aβ clearance in 5xFAD mice [56]. Interestingly, we detected an elevated level of soluble Aβ in 5xFAD/AQP4 KO mice older than 24 weeks (Fig. 5). We saw that transcription of AQP4 was upregulated starting from 24 weeks of age, and it increased in an age-dependent manner (Fig. 3). The accumulation of AQP4 protein was found around amyloid plaques in 5xFAD mice (Fig. 3). These observations suggest that the ectopic expression of AQP4 is required for the maintenance of soluble Aβ level in parenchyma. It should be noted that we also detected an elevated level of soluble Aβ in 5xFAD/AQP4 KO mice aged 8 weeks (Fig. 5), which is a stage in which amyloid plaques are barely detectable. At this stage, the amount of amyloid plaques might not be high enough to capture the produced Aβ, supporting the idea that when the capacity of plaques to incorporate Aβ is not high enough, soluble Aβ level is elevated. Thus, an elevated level of soluble Aβ is one potential factor responsible for the accelerated loss of mobility and the aggravation of epileptiform neuronal activity/convulsions observed in 5xFAD/AQP4 KO mice. Cirrito et al. suggested that soluble Aβ in tissue lysates contains not only interstitial soluble Aβ but also Aβ loosely associated to amyloid plaques solubilized after tissue lysis, even in mild buffer, and thus, soluble Aβ in tissue lysates is not truly soluble and diffusible in the extracellular space [7]. We detected increased level of interstitial Aβ in 5xFAD/AQP4 KO females older than 25 weeks old as compared with age-matched 5xFAD females, although there was no significant difference between these groups. Insoluble Aβ levels in tissue lysates in these two groups are almost the same. Nevertheless, Soluble Aβ levels in tissue lysates were high in 5xFAD/AQP4 KO females. Therefore, it is possible that in 5xFAD/AQP4 KO females, the increased levels of soluble Aβ reflect increase in the amount of Aβ loosely associated to amyloid plaques because of lack of AQP4 solubilized after tissue lysis, which is consistent with Smith et al. [49]. It is also possible that the clearance of neurotoxic substances derived from degenerating neurons, including lipids, nucleotides, proteins, neurotransmitters, and ions, is impaired in 5xFAD/AQP4 KO mice. The expression of TREM2 was upregulated from 32 weeks of age (Fig. 6). Therefore, it is possible that neurodegeneration progressively occurred at this stage. Further investigations are required to elucidate the role of AQP4 in the abnormal behavior and aggravation of epileptiform neuronal activity/convulsions, which occur after amyloid plaque deposition and neuroinflammation.

## Conclusions

Using 5xFAD mice, we examined the effects of deleting AQP4 on the pathogenesis of AD after the accumulation of amyloid plaques. AQP4 was found to not be directly involved in gliosis, neuroinflammation in response to amyloid plaque deposition, or cognitive function determined by T-maze test. However, we found a neuroprotective function of AQP4 against other age-dependent deterioration of neuronal function progressing independently of amyloid plaque deposition and following gliosis, which include a reduction in nighttime movement associated with epileptiform activity of the brain and convulsions. Our results provide an important perspective for developing new diagnostic methods and treatments for Alzheimer’s disease.

## Supplementary information

**Additional file 3.** Supplemental Movie S3.

**Additional file 4.** Supplemental Movie S4.

**Additional file 5.** Supplemental Movie S5.

**Additional file 6.** Supplemental Movie S6.

**Additional file 7.** Supplemental Movie S7.

**Additional file 8.** Supplemental Movie S8.

**Additional file 9.** Supplemental Movie S9.

**Additional file 10.** Supplemental Movie S10.

**Additional file 11.** Supplemental Figures.

**Additional file 12.** Supplemental Tables.

## Data Availability

The datasets used and/or analyzed during the current study available from the authors on reasonable request.

## Abbreviations

Aβ: Amyloid β
AD: Alzheimer’s disease
APP: Amyloid precursor protein
AQP4: Aquaporin-4
CAA: Cerebral amyloid angiopathy
CNS: Central nervous system
CSF: Cerebrospinal fluid
CUBIC: Clear, Unobstructed Brain/Body Imaging Cocktails
CX3CR1: CX3C chemokine receptor 1
EAE: Experimental autoimmune encephalomyelitis
EEG: Electroencephalography/electroencephalogram
FAD: Familial Alzheimer’s disease
GFAP: Glial fibrillary acidic protein
Iba1: Ionized calcium binding adaptor molecule 1
ISF: Interstitial fluid
KO: Knockout
LPS: Lipopolysaccharide
PFA: Paraformaldehyde
PS1: Presenilin 1
TREM2: triggering receptor expressed on myeloid cell 2
