# Supplementary material for: Behavioral and electrophysiological evidence for a neuroprotective role of aquaporin-4 in the 5xFAD transgenic mice model
Source: Acta Neuropathol Commun. 2020 May 12;8:67. doi: 10.1186/s40478-020-00936-3 (PMC7218576; doi:10.1186/s40478-020-00936-3)
Supplement: Supplementary file 11 — Additional file 11. Supplemental Figures. [file 40478_2020_936_MOESM11_ESM.pdf]

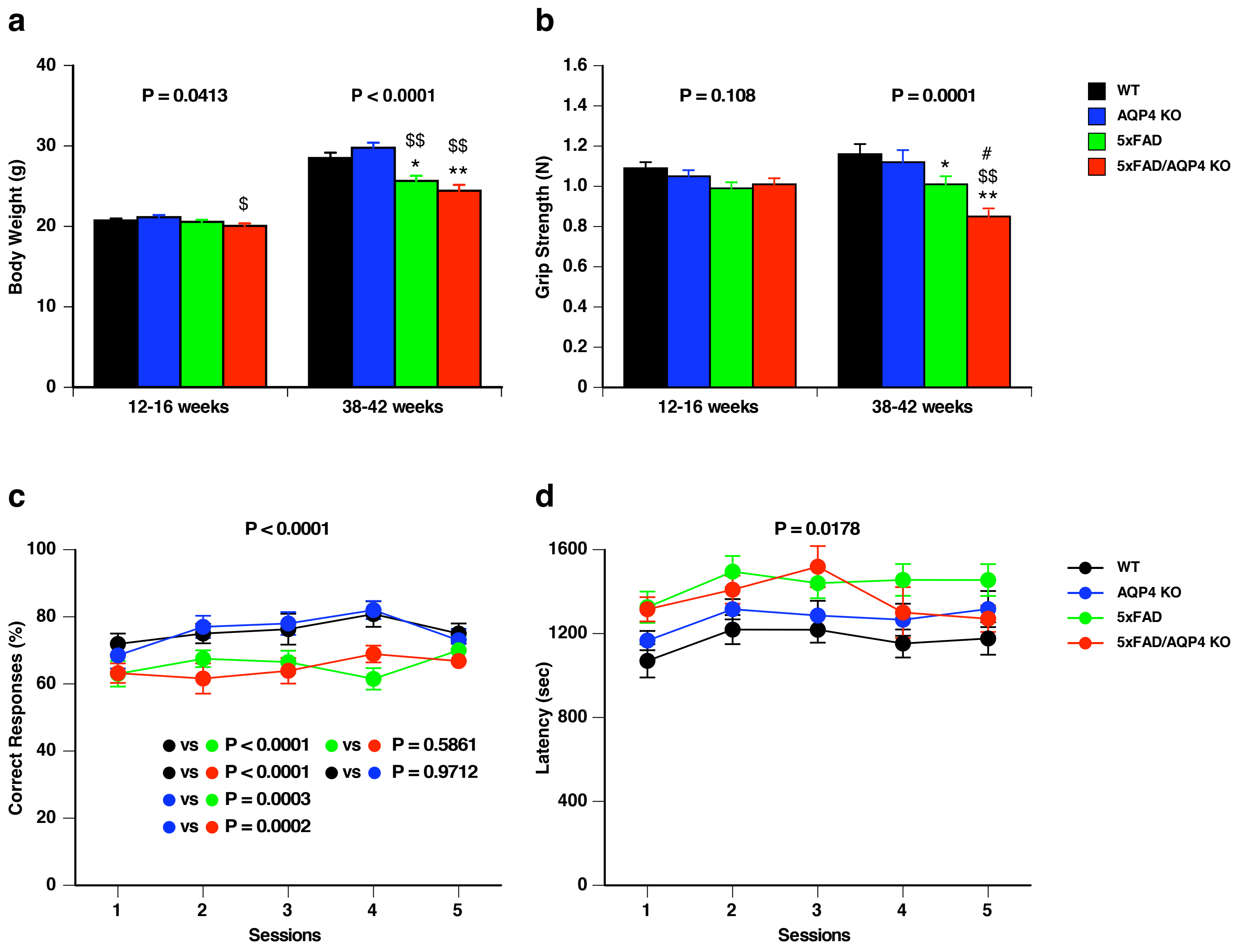


**Supplemental Fig. S1** Behavioral tests of 5xFAD mice. (**a**) Body weight. The p values indicate genotype effect in one-way ANOVA. * ($P$=0.0042) and ** ($P$=0.0001) represent significant differences versus wild type, and $ (0.0048) and $$ ($P$<0.0001) represents significant difference versus AQP4 KO determined by Fisher's Protected Least Significant Difference test. (**b**) Grip strength. The p values indicate genotype effect in one-way ANOVA. * ($P$=0.0321) and ** ($P$=0.0001) represent significant differences versus wild type, $$ ($P$=0.0002) represents significant difference versus AQP4 KO, and # ($P$=0.0262) represents significant difference versus 5xFAD determined by Fisher's Protected Least Significant Difference test. (**c, d**) T-maze test: Percentage of correct responses (**c**) and latency (**d**) were presented. Values are mean ± S.E.M of 16-20 animals.

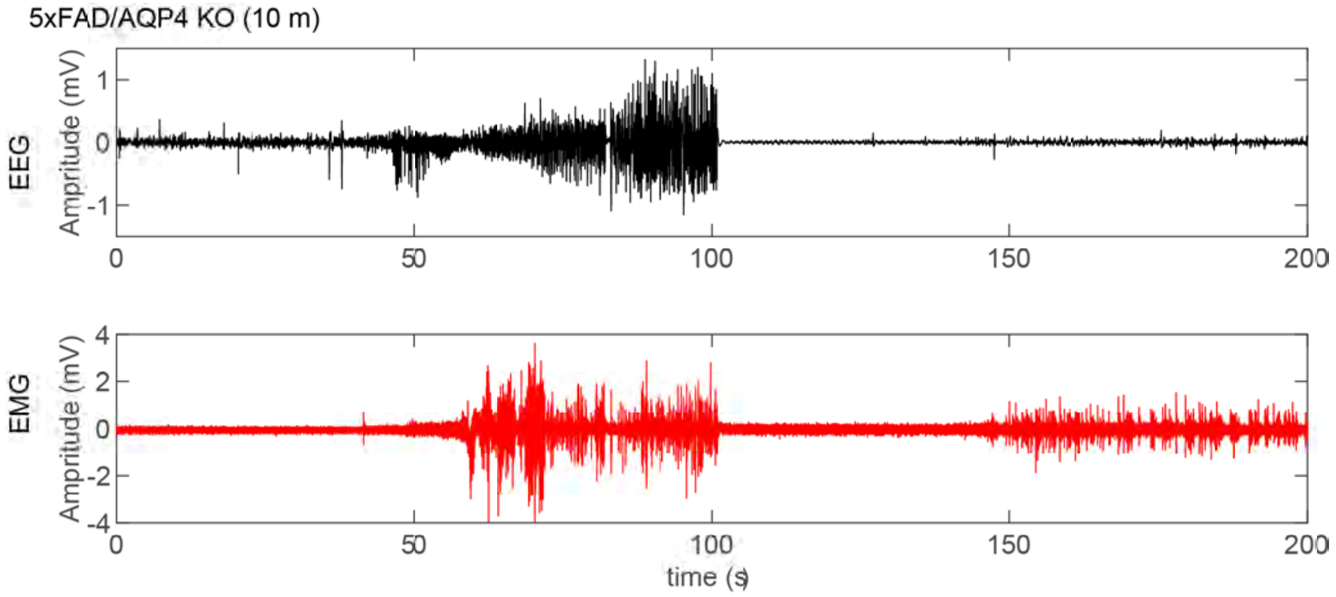


**Supplemental Fig. S2** Detection of a seizure in a 5xFAD/AQP4 KO mouse during EEG recording. (**a**) EEG (black) and electromyogram (EMG) (red) recordings where an epileptic discharge was seen around50-100 sec. Behavior of the mouse druing the recording is shown in Supplemental Movie S2

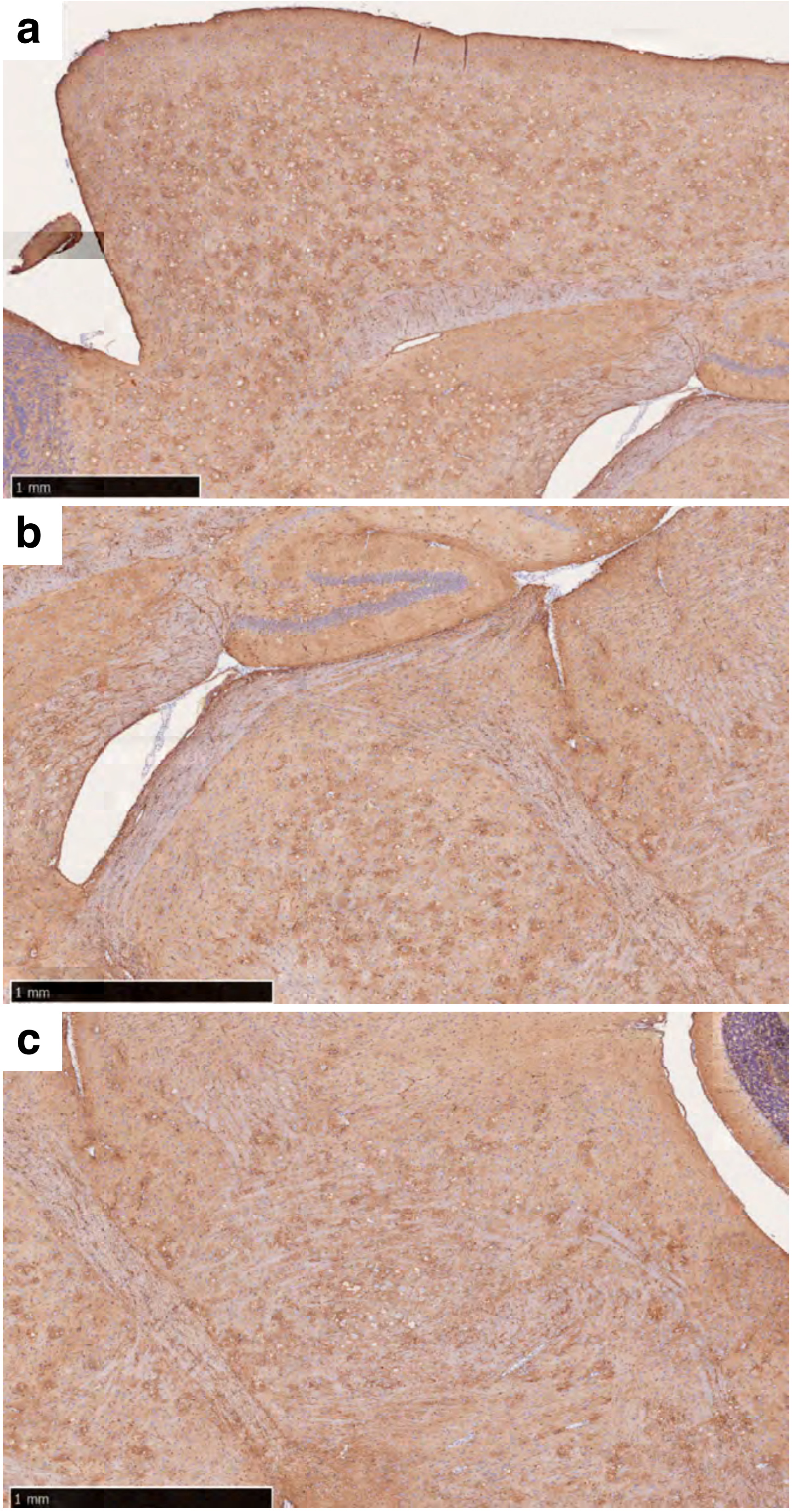


**Supplemental Fig. S3** Expression of AQP4 in the brain of a 5xFAD mouse. Immunostaining of AQP4 in the cortex (**a**), hippocampus and thalamus (**b**), and brain stem (**c**) of a 40-week-old female 5xFAD mouse. Scale bars = 1 mm.

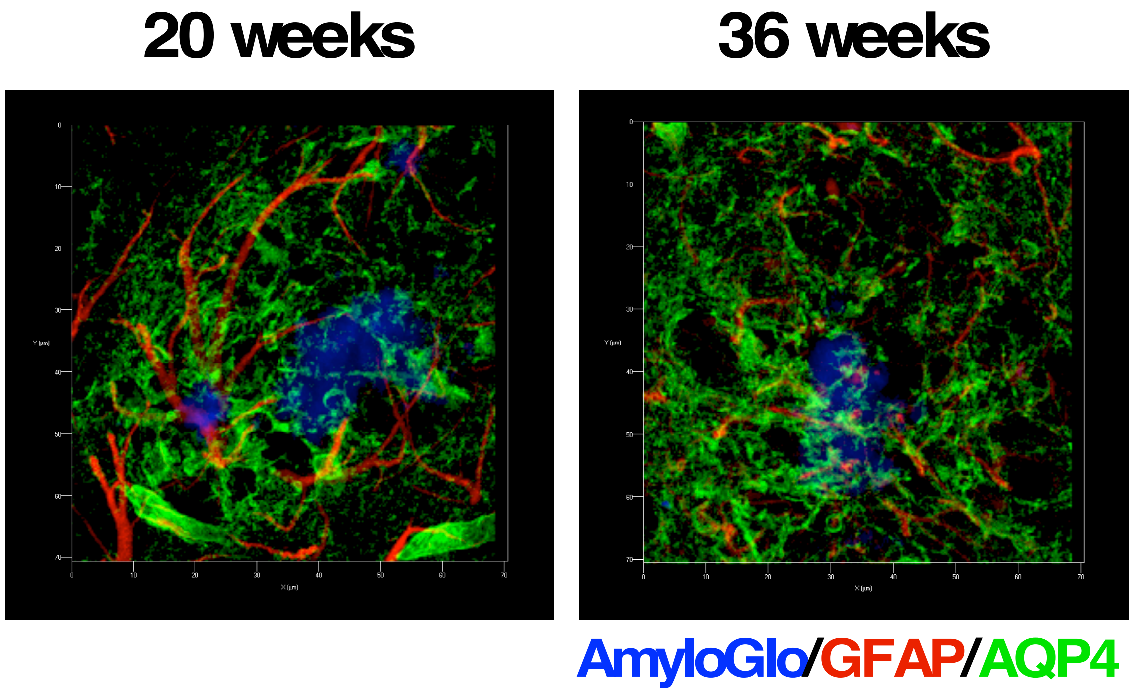


**Supplemental Fig. S4** Expression of AQP4 in the cortical region of a 5xFAD mouse. One-hundred-μm sections from 20-week-old (left) or 36-week-old (right) female 5xFAD mouse were cleared using the CUBIC method and stained with AmyloGlo (blue), GFAP (red), and AQP4 (green). Z-stack images of the cortical region were obtained using laser scanning confocal microscope. Each focal plane of the z-stack images is shown in Supplemental Movies S3, and S4 for a brain from 20-week-old and 36-week-old female mice, respectively. Thickness of a single focal plane is 0.32 μm.

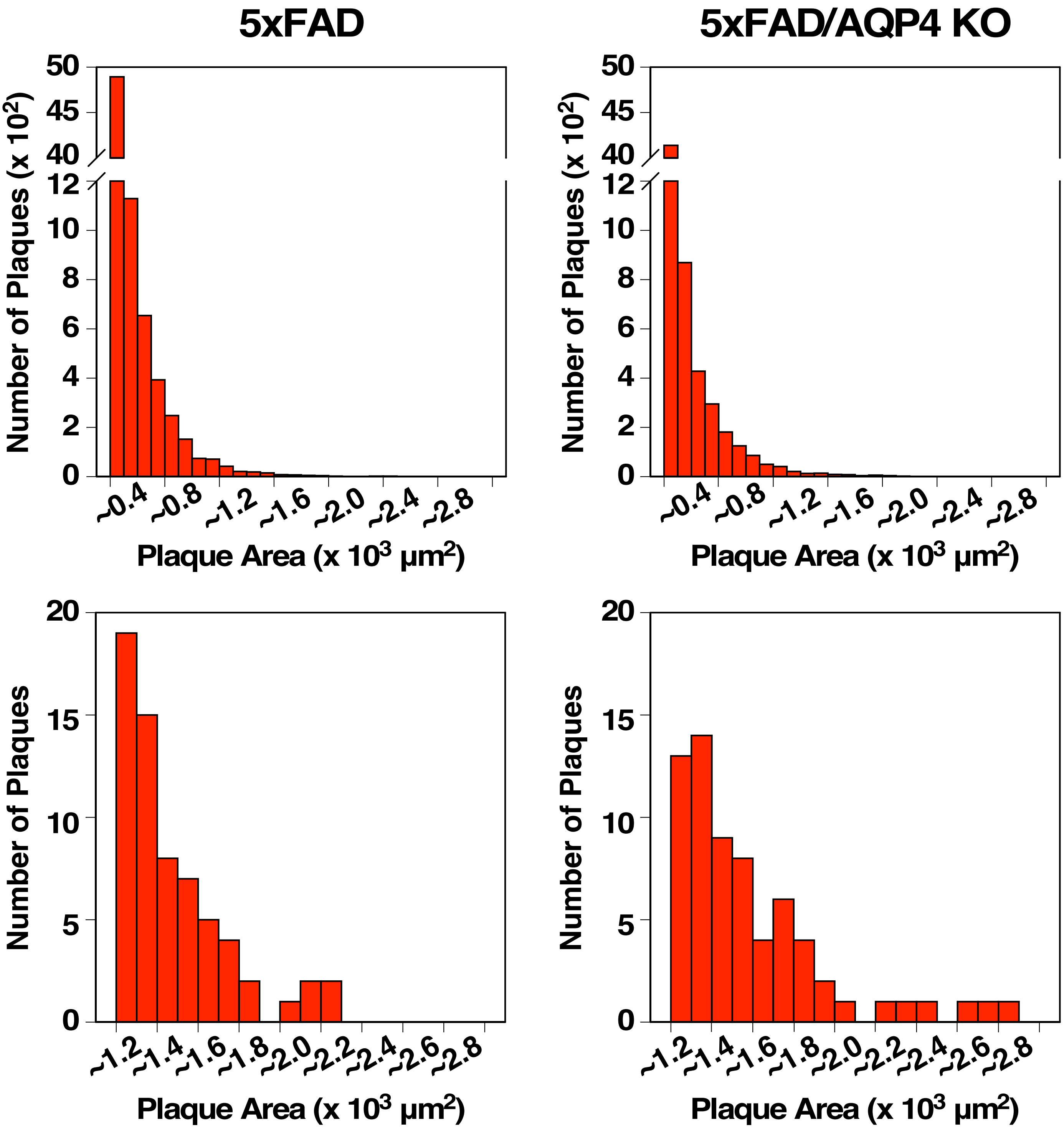


**Supplemental Fig. S5** Histogram of the size of amyloid plaques stained with an anti-A$\beta_{42}$ antibody. Amyloid plaques in the cerebral cortex of 5xFAD (left panels) and 5xFAD/AQP4 KO (right panels) mice were counted using ImageJ. Plaques with an area greater than 1000 $\mu m^2$ in the upper panels were plotted in the lower panels.

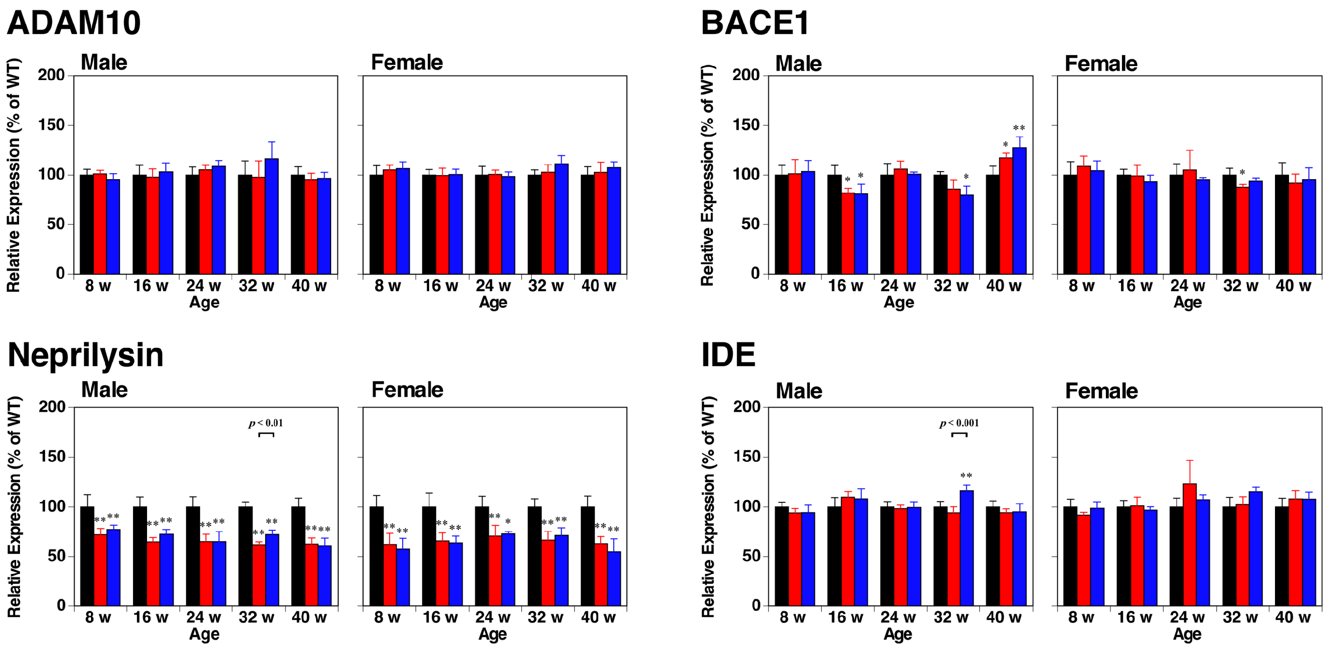


**Supplemental Fig. S6** Expression of Aβ-producing and degrading enzymes in 5xFAD mice. qPCR analysis of brain hemispheres from wild-type (black columns), 5xFAD (red columns), and 5xFAD/AQP4 KO (blue columns) mice. The level of the transcript of each gene in 5xFAD and 5xFAD/AQP4 KO mice was determined as a % of that of age-matched wild-type mice. Values are mean ± S.E.M of 5-6 individuals. * (P<0.01) and ** (P<0.001) represent significant differences versus wild-type mice. The primers used are listed in Supplemental Table S2.

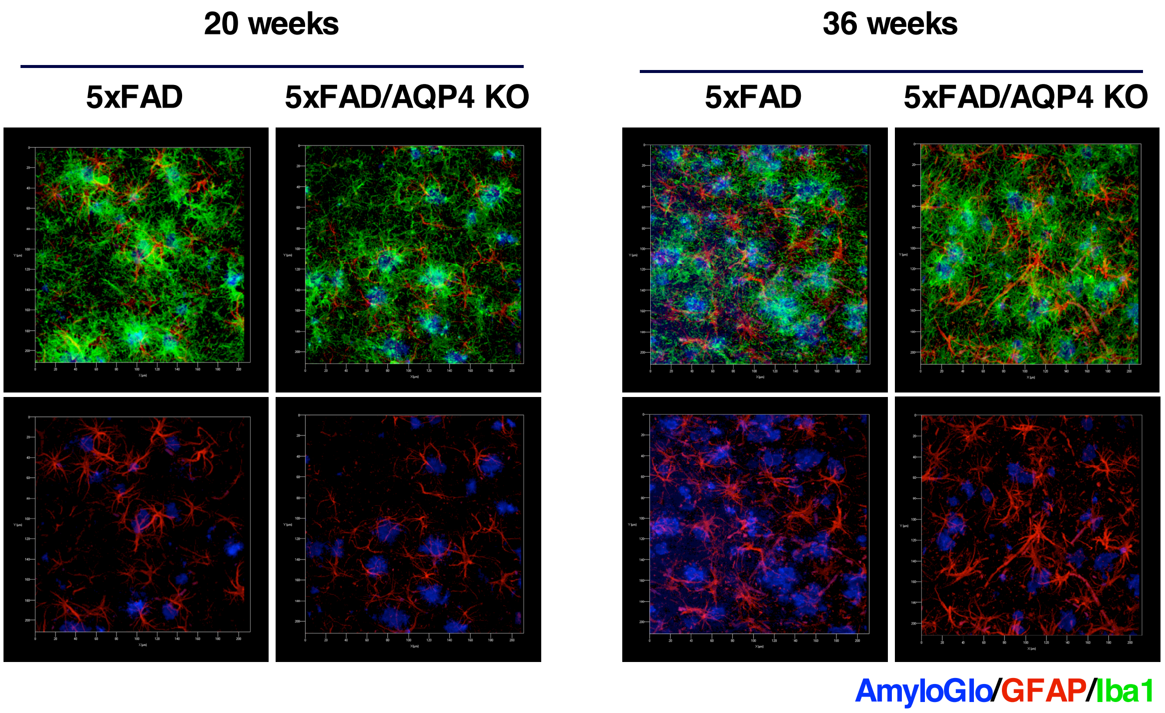


**Supplemental Fig. S7** Accumulation of reactive astrocytes and activated microglia around amyloid plaques. One-hundred-μm sections from 20 week-old 5xFAD, 20 week-old 5xFAD/AQP4 KO, 36 week-old 5xFAD, and 36 week-old 5xFAD/AQP4 KO mice were cleared using the CUBIC method and stained with AmyloGlo (blue), GFAP (red), and Iba1 (green). Z-stack images of the cortical region were obtained using laser scanning confocal microscope. The bottom pannels are the same images as top pannels without Iba1 signals. Each focal plane of the z-stack images is shown in Supplemental Movies S7, S8, S9, and S10 for brains from 20-week-old 5xFAD, 20-week-old 5xFAD/AQP4 KO, 36-week-old 5xFAD, and 36-week-old 5xFAD/AQP4 KO mice, respectively. Thickness of a single focal plane is 0.32 μm.
