# Supplementary material for: Behavioral and electrophysiological evidence for a neuroprotective role of aquaporin-4 in the 5xFAD transgenic mice model
Source: Acta Neuropathol Commun. 2020 May 12;8:67. doi: 10.1186/s40478-020-00936-3 (PMC7218576; doi:10.1186/s40478-020-00936-3)
Supplement: Supplementary file 12 — Additional file 12. Supplemental Tables. [file 40478_2020_936_MOESM12_ESM.pdf]

Supplemental Table S1. Numbers of mice used in this study

Figures	WT											
	long term	8 w		16 w		24 w		32 w		40 w		48 w
	F	M	F	M	F	M	F	M	F	M	F	F
Fig. 1												
Fig. 2, Supplemental Fig. S2												1
Fig. 3a, Fig. 4m-n, Fig. 5a-b, Figt 6a, Supplemental Fig. S6		5	6	6	6	6	5	6	6	6	6	
Fig. 3b-e, Fig. 4a-j, Fig. 6b-g Supplemental Fig. S3, S5											1	
Fig. 4k-l												
Fig. 5c-f												
Supplemental Fig. S1	16											
Supplemental Fig. S4, S7												

Figures	AQP4 KO	
	long term	48 w
	F	F
Fig. 1	11	
Fig. 2, Supplemental Fig. S2		1
Fig. 3a, Fig. 4m-n, Fig. 5a-b, Figt 6a, Supplemental Fig. S6		
Fig. 3b-e, Fig. 4a-j, Fig. 6b-g Supplemental Fig. S3, S5		
Fig. 4k-l		
Fig. 5c-f		
Supplemental Fig. S1	20	
Supplemental Fig. S4, S7		

Figures	5xFAD													
	long term	8 w		16 w		20 w	24 w		32 w		36 w	40 w		45 w
	F	M	F	M	F	F	M	F	M	F	F	M	F	F
Fig. 1	11													
Fig. 2, Supplemental Fig. S2					5								6	
Fig. 3a, Fig. 4m-n, Fig. 5a-b, Figt 6a, Supplemental Fig. S6		6	6	6	6		6	5	6	6 (2)*		6	6	
Fig. 3b-e, Fig. 4a-j, Fig. 6b-g Supplemental Fig. S3, S5													3	
Fig. 4k-l														3
Fig. 5c-f								9**		7**				
Supplemental Fig. S1	20													
Supplemental Fig. S4, S7						2					2			

Figures	5xFAD/AQP4 KO													
	long term	8 w		16 w		20 w	24 w		32 w		36 w	40 w		45 w
	F	M	F	M	F	F	M	F	M	F	F	M	F	F
Fig. 1	11													
Fig. 2, Supplemental Fig. S2					5								10	
Fig. 3a, Fig. 4m-n, Fig. 5a-b, Figt 6a, Supplemental Fig. S6		6	6	6	6		6	5	6	6 (1)*		6	6	
Fig. 3b-e, Fig. 4a-j, Fig. 6b-g Supplemental Fig. S3, S5													3	
Fig. 4k-l														3
Fig. 5c-f								9**		7				
Supplemental Fig. S1	20***													
Supplemental Fig. S4, S7						2					2			

*Numbers in parentheses are individuals added for measurement of soluble and insoluble Aβ. **$A\beta_{40}$ were not measured for one individual due to a shortage of the sample. ***Eighteen mice were used for the measurement of body weight and grip strength at 38-42 weeks of age due to death of two individuals.

Supplemental Table S2. Primers used for qPCR analysis

Genes	Sequence
Total AQP4	5'-AACCTCACCGCTGGCCATGGGCTCCTG-3'<br>5'-TACGGAAGACAATACCTCTCCCGAAGAGTC-3'
GAPDH	5'-ACTGGTGTCTTCACCACCATGGAGAAGGC-3'<br>5'-CATGAGGTCCACCACCCTGTTGCTGTAGC-3'
GFAP	5'- CTCGGCACCCTGAGGCAGAAGCTCCAAGATGAAAC-3'<br>5'- CTTAGACCGATACCACTCCTCTGTCTCTTGCATG-3'
TREM2	5'- ACCTCTCCACCAGTTTCTCCTGCTGCTGATCACAG-3'<br>5'- AGTACTTTCTGCAGGACCTCAGCCTCTCGGCCTCG-3'
CX3CR1	5'-CGTGAGACTGGGTGAGTGACTGGCACTTCCTGCAG-3'<br>5'- CAATGAAGAAGAAGGCAGTCGTGAGCTTGCACATG-3'
IL1-β	5'-AGGCCTAATAGGCTCATCTGGGATCCTCTC-3'<br>5'- GTTCATCTCGGAGCCTGTAGTGCAGTTGTC-3'
IL6	5'- TTCTTGGGACTGATGCTGGTGACAACCACG-3'<br>5'- AGGTAGCTATGGTACTCCAGAAGACCAGAG-3'
ADAM10	5'- AGCATGCTGCTAGTAGTGGTCCTGAGCTCCTGAG-3'<br>5'- GATGCCTGTGTTCAATGACTTCTTCTTGCCATCTG-3'
BACE1	5'- AGAGGCAGCTTTGTGGAGATGGTGGACAACCTGAG-3'<br>5'- ATGTTGGGAATGTGGGTCTGCTTCACCAGGGAGTC-3'
NEP	5'- CAGCCCCCATTCTTCAGTGCTCAGCAATCCAACTC-3'<br>5'- ATGTTCAGTATCTTGTGAAGATCACCAAACCCGAC-3'
IDE	5'- CACCCCACTACCTGGAGAGCAGAGTAGAAGCCTTC-3'<br>5'- CAGTCATGTTGTGAATCACTTCAGGTTGTGGCAAG-3'
